# Utilization of outpatient services in refugee settlement health facilities: a comparison by age, gender, and refugee versus host national status

**DOI:** 10.1186/1752-1505-5-19

**Published:** 2011-09-21

**Authors:** William M Weiss, Alexander Vu, Hannah Tappis, Sarah Meyer, Christopher Haskew, Paul Spiegel

**Affiliations:** 1 Department of International Health, Johns Hopkins Bloomberg School of Public Health, 615 N. Wolfe Street, Baltimore, Maryland, 21205, USA; 2 Department of Emergency Medicine, Johns Hopkins School of Medicine, Baltimore, Maryland 21205, USA; 3 United Nations High Commissioner for Refugees, Geneva, Switzerland

## Abstract

**Background:**

Comparisons between refugees receiving health care in settlement-based facilities and persons living in host communities have found that refugees have better health outcomes. However, data that compares utilization of health services between refugees and the host population, and across refugee settlements, countries and regions is limited. The paper will address this information gap. The analysis in this paper uses data from the United Nations High Commissioner of Refugees (UNHCR) Health Information System (HIS).

**Methods:**

Data about settlement populations and the use of outpatient health services were exported from the UNHCR health information system database. Tableau Desktop was used to explore the data. STATA was used for data cleaning and statistical analysis. Differences in various indicators of the use of health services by region, gender, age groups, and status (host national vs. refugee population) were analyzed for statistical significance using generalized estimating equation models that adjusted for correlated data within refugee settlements over time.

**Results:**

Eighty-one refugee settlements were included in this study and an average population of 1.53 million refugees was receiving outpatient health services between 2008 and 2009. The crude utilization rate among refugees is 2.2 visits per person per year across all settlements. The refugee utilization rate in Asia (3.5) was higher than in Africa on average (1.8). Among refugees, females have a statistically significant higher utilization rate than males (2.4 visits per person per year vs. 2.1). The proportion of new outpatient attributable to refugees is higher than that attributable to host nationals. In the Asian settlements, only 2% outpatient visits, on average, were attributable to host community members. By contrast, in Africa, the proportion of new outpatient (OPD) visits by host nationals was 21% on average; in many Ugandan settlements, the proportion of outpatient visits attributable to host community members was higher than that for refugees. There was no statistically significant difference between the size of the male and female populations across refugee settlements. Across all settlements reporting to the UNHCR database, the percent of the refugee population that was less than five years of age is 16% on average.

**Conclusions:**

The availability of a centralized database of health information across UNHCR-supported refugee settlements is a rich resource. The SPHERE standard for emergencies of 1-4 visits per person per year appears to be relevant for Asia in the post-emergency phase, but not for Africa. In Africa, a post-emergency standard of 1-2 visits per person per year should be considered. Although it is often assumed that the size of the female population in refugee settlements is higher than males, we found no statistically significant difference between the size of the male and female populations in refugee settlements overall. Another assumption---that the under-fives make up 20% of the settlement population during the emergency phase---does not appear to hold for the post-emergency phase; under-fives made up about 16% of refugee settlement populations.

## Background

The global estimate number of people who are forcibly displaced is 43.3 million at the end of 2009. Included in this population are 15.2 million refugees, of whom 10.4 million fall under mandate of the United Nations High Commissioner of Refugees [1]. Less than half of the refugees live in settlement facilities. Comparisons between refugees receiving health care in settlement-based facilities and persons living in host communities have found that refugees have better health outcomes [2]. Improved access to health services is attributed to lower neonatal mortality rates and maternal mortality among the refugees when compared to the host population in certain settings [3,4]. However, data comparing utilization of health services between refugees and the host population, and across refugee settlements, countries and regions is limited. The paper will compare the use of outpatient health services by age and gender, and between refugees and host populations.

The analysis in this paper uses data from the United Nations High Commissioner of Refugees (UNHCR) Health Information System (HIS). This HIS is a standardized tool used by UNHCR and its partners to public health programs delivered to populations of concern [5]. The aim has been to improve the health status of refugees and other displaced persons through evidence-based policy formulation, better management of health programs, and ultimately actions that improve refugee health. In August 2010, a total of 20 operations in Africa, Asia and Middle East and North Africa regions were reporting into the HIS using common tools and guidelines. The total population under surveillance was approximately 1.5 million refugees in 102 refugee sites and across 25 different partners.

## Methods

Data about settlement populations and the use of outpatient health services were exported from the UNHCR health information system database. The data included settlement specific information about the following: month of report, total settlement population and population size by gender and age group (less than five years of age, five years of age and older). Outpatient health services data included the total number of new outpatient visits (for all causes) and a breakdown of this data by region, country, settlement, month, gender, and status (refugee versus host national). We also had data about outpatient diagnoses and a breakdown by region, country, settlement, month, age and gender. Information about use of settlement outpatient services was combined with population data to calculate utilization rates and proportions where possible. Note that population denominators were not available for information about use of settlement outpatient department (OPD) services by host nationals. Instead, we collected information on national estimates of the female and less than five years of age populations [6].

Tableau Desktop was used to explore the data [7]. STATA was used for data cleaning and statistical analysis [8]. Differences in various indicators of the use of health services by region, gender, age groups, and status (host national vs. refugee population) were analyzed for statistical significance using generalized estimating equation models that adjusted for correlated data within settlements over time.

## Results

Table 1 shows the distribution of settlement reports by region and country. A significant majority of monthly settlement reports came from the African region. The number of settlements per country varied widely from one (Cameroon, Djibouti, Yemen) to 15 (Chad). In total, 81 settlements were included in this study and an average population of 1.53 million refugees was receiving outpatient health services between 2008 and 2009.

**Table 1:** Countries represented in the analysis by Region, Number of Camps Reporting to the UNHCR Health Information System, and Average Number of Refugees Served each Month, 2008-09*

Region	Host Country	Number of Camps	Avg Monthly Population Served
**Asia**	Bangladesh	2	28,048
	Nepal	7	100,525
	Thailand	9	198,098
Sub-Total		18	326,671
			
**Africa**	Burundi	4	19,546
	Cameroon	1	3,871
	Chad	15	257,526
	Djibouti	1	8,688
	Ethiopia	5	72,020
	Guinea	1	3,341
	Kenya	4	289,861
	Rwanda	3	50,365
	Sudan	8	98,714
	Tanzania	5	198,098
	Uganda	11	144,309
	Yemen	2	12,115
	Zambia	4	49,707
Sub-Total		65	1,246,118

**Total**		**81**	**1,534,832**

* Countries were excluded if camps were piloting the UNHCR HIS, or where there were fewer than six monthly reports total for the two-year period for the country

### Outpatient Utilization Rates for Refugee Populations

The mean number of visits per refugee per year is displayed in Table 2. On a monthly basis, refugee settlements report the number of new outpatient visits by gender. Using these data, along with population data about females and males, a crude annualized rate of outpatient utilization was calculated along with rates for each gender. Because the UNHCR database does not include information on the size and distribution of the host populations, it was not possible to calculate utilization rates for the host national population.

**Table 2:** Outpatient Department Utilization Rates Per Refugee Per Year by Gender, 2008-2009

		All		Female		Male		M v. F p Value*
			
Region/Country/Camp		Rate/Year *	95% CI*	Rate/Year *	95% CI*	Rate/Year *	95% CI*	
**Africa**		**1.8**	**1.7, 2.0**	**2.0**	**1.8,2.1**	**1.7**	**1.5,1.8**	**< 0.001**
**Burundi**		**4.0**	**3.0, 5.1**	**4.2**	**3.1,5.4**	**3.8**	**2.8,4.8**	**< 0.001**
Bwagiriza		8.4	6.1,10.7	8.8	6.2,11.4	8.0	6.2,9.8	
Gasorwe		2.5	2.3,2.7	2.6	2.3,2.8	2.4	2.1,2.6	
Gihinga		4.3	3.7,4.9	4.7	4.1,5.4	3.8	3.2,4.3	
Musasa		3.7	3.0,4.4	3.8	3.0,4.6	3.6	3.0,4.2	
**Cameroon**	Langui	**3.9**	**3.2,4.5**	**4.1**	**3.4,4.8**	**3.7**	**3.0,4.3**	**< 0.05**
**Chad**		**1.4**	**1.2, 1.6**	**1.6**	**1.2,2.1**	**1.4**	**1.3,1.6**	
Amboko		1.3	0.9,1.6	1.2	0.8,1.6	1.4	0.8,2.0	
Amnabak		0.8	0.6,1.0	0.7	0.6, 0.9	1.0	0.7,1.2	
Bredjing		1.3	1.2,1.4	1.2	1.1,1.4	1.3	1.2,1.4	
Djabal		1.9	1.7,2.1	1.9	1.7,2.1	2.0	1.8,2.1	
Dosseye		2.3	2.0,2.6	2.5	2.2,2.8	2.1	1.8,2.4	
Farchana		1.0	0.8,1.2	0.9	0.7,1.1	1.2	1.0,1.5	
Gaga		1.1	0.9,1.3	1.1	0.9,1.3	1.1	0.9,1.3	
Gondje		0.9	0.6,1.2	0.9	0.6,1.2	0.9	0.6,1.2	
Goz Amer		2.0	1.7,2.2	2.0	1.7,2.2	2.0	1.8,2.2	
Kounoungou		1.1	0.9,1.2	1.0	0.9,1.2	1.1	1.0,1.3	
Mile		1.0	0.9,1.1	1.0	0.9,1.2	1.0	0.9,1.1	
Moula		3.5	3.3,3.6	3.7	3.6,3.9	3.2	3.0,3.4	
Oure Cassoni		1.3	1.1,1.4	1.2	1.1,1.3	1.3	1.2,1.5	
Treguine		1.8	1.6,2.1	4.7	-0.7,10.0	1.9	1.7,2,2	
Yaroungou		0.7	0.5,0.8	0.7	0.5,1.0	0.6	0.5,0.7	
**Djibouti**	Ali Adde	**2.8**	**2.3, 3.2**	**3.1**	**2.6,3.6**	**2.5**	**2.1,2.9**	**< 0.001**
**Ethiopia**		**1.7**	**1.2, 2.1**	**2.0**	**1.4,2.5**	**1.5**	**1.1,1.9**	**< 0.001**
Awbarre		0.9	0.8,1.1	1.1	0.9,1.3	0.8	0.7,0.9	
Fugnido		1.3	1.0,1.5	1.3	1.1,1.6	1.2	0.9,1.5	
Kebribeyah		1.7	1.5,1.9	1.9	1.7,2.0	1.6	1.4,1.7	
Sherkole		1.9	1.0,2.8	2.1	1.2,3.0	1.7	0.9,2.6	
Shimelba		2.7	1.3,4.1	3.6	2.3,4.8	2.3	1.0,3.6	
**Guinea**	Kouankan II	**3.2**	**2.3, 4.0**	**3.4**	**2.4,4.4**	**2.9**	**2.2,3.6**	**< 0.05**
**Kenya**		**1.4**	**1.3, 1.6**	**1.5**	**1.3,1.7**	**1.4**	**1.2,1.5**	**< 0.001**
Dagahaley		1.3	1.2,1.4	1.4	1.2,1.5	1.2	1.1,1.4	
Hagadera		1.1	1.1,1.2	1.2	1.1,1.3	1.1	1.0,1.1	
Ifo		1.3	1.2,1.4	1.4	1.3,1.5	1.3	1.1,1.5	
Kakuma		1.9	1.6,2.1	2.0	1.8,2.3	1.8	1.5,2.0	
**Rwanda**		**1.7**	**1.1, 2.4**	**1.7**	**1.1,2.3**	**1.7**	**1.0,2.4**	
Gihembe		1.3	1.0,1.6	1.3	1.0,1.6	1.3	1.0,1.6	
Kiziba		1.0	0.9,1.2	1.1	1.0,1.2	1.0	0.8,1.1	
Nyabiheke		3.0	2.1,4.0	2.9	2.1,3.8	3.2	2.1,4.4	
**Sudan**		**2.1**	**1.6, 2.6**	**2.4**	**1.9,2.8**	**1.8**	**1.4,2.3**	**< 0.001**
Abuda		2.7	2.3,3.0	3.3	2.8,3.9	3.2	2.1,4.4	
Fau 5		4.5	3.5,5.5	4.5	3.6,5.5	4.3	3.3,5.3	
Girba		1.7	1.6,1.8	1.9	1.8,2.1	1.4	1.3,1.5	
Kilo 26		1.8	1.5,2.0	2.2	1.9,2.5	1.5	1.3,1.6	
Shagarab I II III		1.8	1.6,2.0	2.2	1.8,2.6	1.5	1.4,1.6	
Suki		2.6	2.3,2.8	3.0	2.7,3.2	2.2	2.0,2.5	
Um Gargour		0.9	0.7,1.1	1.2	0.8,1.5	0.8	0.6,1.0	
Wad Sharifey		1.3	1.1,1.5	1.3	1.1,1.5	1.2	1.0,1.5	
**Tanzania**		**2.6**	**2.2, 3.0**	**2.7**	**2.3,3.2**	**2.4**	**2.1,2.7**	**< 0.001**
Lugufu		2.2	1.9,2.5	2.1	1.8,2.5	2.2	1.9,2.6	
Lukole		3.3	2.3,4.2	3.7	2.6,4.9	2.9	2.1,3.6	
Mtabila		2.8	2.5,3.1	3.1	2.7,3.4	2.5	2.3,2.8	
Nduta		3.4	2.3,4.4	3.6	2.5,4.8	3.1	2.1,4.1	
Nyarugusu		1.9	1.4,2.4	1.9	1.4,2.5	1.9	1.3,2.4	
**Uganda**		**1.2**	**1.0, 1.4**	**1.4**	**1.2,1.6**	**1.0**	**0.9,1.2**	**< 0.001**
Adjumani		0.9	0.7,1.0	1.0	0.9,1.1	0.7	0.6,0.8	
Ikafe		0.8	0.6,0.9	1.0	0.7,1.3	0.6	0.5,0.7	
Imvepi		0.8	0.4,1.1	0.8	0.5,1.0	0.8	0.4,1.2	
Kiryandongo		1.5	1.0,2.0	1.7	1.2,2.3	1.3	0.8,1.7	
Kyaka II		1.1	0.9,1.3	1.2	1.0,1.4	1.0	0.8,1.2	
Kyangwali		1.3	1.2,1.5	1.5	1.3,1.7	1.1	1.0,1.2	
Madi Okollo		0.8	0.7,1.0	0.9	0.7,1.1	0.7	0.6,0.9	
Nakivale		1.2	0.9,1.5	1.3	1.0,1.6	1.2	0.9,1.6	
Oruchinga		2.1	1.3,3.0	2.5	1.6,3.5	1.8	1.0,2.5	
Palorinya		1.5	1.1,1.9	1.8	1.5,2.1	1.2	0.7,1.6	
Rhino		0.8	0.3,1.3	1.0	0.4,1.6	0.7	0.3,1.1	
**Yemen**	Kharaz	2.1	1.3,2.8	2.1	1.3,3.0	2.0	1.4,2.7	
**Zambia**		**1.6**	**1.2, 2.1**	**1.8**	**1.3,2.2**	**1.5**	**1.1,1.9**	**< 0.001**
Kala		1.0	0.8,1.2	0.9	0.8,1.2	1.0	0.8,1.2	
Maheba		2.1	1.0,3.2	2.3	1.1,3.6	1.8	0.9,2.8	
Mayukwayukwa		1.2	1.0,1.3	1.4	1.2,1.6	1.0	0.8,1.1	
Mwange			2.3	1.9,2.7		2.2	1.8,2.5	
**Asia**		**3.5**	**3.3, 3.7**	**3.8**	**3.6,4.0**	**3.2**	**3.0,3.4**	**< 0.001**
**Bangladesh**		**4.1**	**3.2, 4.9**	**4.2**	**3.2,5.2**	**3.9**	**3.1,4.7**	**< 0.05**
Kutupalong		5.0	4.2,5.7	5.1	4.2,6.1	4.7	4.1,5.4	
Nayapara		3.2	2.9,3.4	3.3	3.1,3.6	3.0	2.8,3.3	
**Nepal**		**3.5**	**3.3, 3.8**	**3.9**	**3.6,4.1**	**3.2**	**2.9,3.4**	**< 0.001**
Beldangi I		3.0	2.5,3.4	3.2	2.8,3.7	2.7	2.3,3.1	
Beldangi II		3.1	2.5,3.6	3.4	2.8,4.0	2.8	2.2,3.3	
Beldangi II ext		3.4	2.8,3.9	3.7	3.1,4.3	3.0	2.5,3.5	
Goldhap		4.4	3.7,5.2	4.9	4.1,5.7	4.0	3.3,4.7	
Khudunabari		3.5	3.0,3.9	3.8	3.4,4.2	3.1	2.7,3.6	
Sanishare		3.4	3.0,3.8	3.7	3.3,4.1	3.1	2.7,3.5	
Timai		4.0	3.4,4.5	4.3	3.7,4.9	3.6	3.1,4.2	
**Thailand**		**3.4**	**3.1, 3.7**	**3.7**	**3.3,4.0**	**3.1**	**2.9,3.4**	**< 0.001**
Ban Don Yang		3.8	3.5,4.1	4.1	3.7,4.4	3.6	3.3,3.8	
Ban Mae Surin		5.3	4.5,6.0	5.8	5.0,6.6	4.8	4.1,5.5	
Ban Mai Nai Soi		3.2	2.9,3.5	3.3	3.0,3.5	3.1	2.8,3.4	
Mae La		2.4	2.1,2.7	2.4	2.2,2.7	2.3	2.0,2.7	
Mae La Oon		3.5	3.3,3.8	3.6	3.3,4.0	3.4	3.1,3.8	
Mae Ra Ma Luang		3.9	3.6,4.1	4.2	4.0,4.5	3.5	3.3,3.7	
Nu Poh		2.5	2.4,2.6	2.8	2.6,2.9	2.2	2.1,2.3	
Tham Hin		3.5	3.2,3.9	3.9	3.5,4.3	3.2	2.9,3.5	
Umpiem Mai		2.5	2.3,2.6	2.8	2.6,3.0	2.1	2.0,2.3	
**All Regions**		**2.2**	**2.0,2.4**	**2.4**	**2.3,2.6**	**2.1**	**1.9,2.2**	**< 0.001**
**Asia - Africa Differential**		**1.7**	**1.4, 2.0**	**1.8**	**1.6,2.1**	**1.6**	**1.3,1.8**	
		**(p < 0.001)**		**(p < 0.001)**		**(p < 0.001)**		

* Values, Confidence Intervals and Significance are based on Generalized Estimating Equations, population-averaged model (Std. Err. adjusted for clustering on Camp)

#### Crude OPD utilization rates among refugee populations

The crude utilization rate is 2.2 visits per person per year across all settlements. The outpatient utilization rate in Asia (3.5) was higher than in Africa on average (1.8). In most settlements across countries refugees were utilizing outpatient services at the SPHERE standard of 1.0 to 4.0 visits per person per year for displaced populations in emergencies [9]. A few settlements utilization rates greater than 4.0 (e.g., Bwagiriza settlement in Burundi, Kutupalong settlement in Bangladesh, and Ban Mae Surin settlement in Thailand). And, some settlements had utilization rates lower than 1.0 (i.e., Yaroungou settlement in Chad, Madi Okollo settlement in Uganda).

#### Gender differences in OPD utilization rates among refugee populations

Across refugee settlements reporting to the UNHCR database, females have a statistically significant higher utilization rate than males (2.4 visits per person per year vs. 2.1). This pattern is seen in all regions. In Africa, utilizations rates for females averaged 2.0 visits per person per year compared to 1.7 for males. In Asia, female utilization rates averaged 3.8 vs. 3.2 for males. Average utilization rates for both males and females fall within the SPHERE standard of 1.0 - 4.0 visits per person per year for emergencies.

### Proportion of New Outpatient Visits per Month by Status and Gender

#### New OPD visits per month by status

Table 3 shows the mean proportion of new visits in a month attributable to refugees versus host nationals. The proportion of new outpatient visits to settlement health facilities attributable to refugees is higher than that attributable to host nationals. In the Asian settlements, refugees accounted for about 98% of outpatient visits. Only 2% outpatient visits, on average, were attributable to host community members. By contrast, in Africa, the proportion of new outpatient (OPD) visits by refugees and host nationals was 79% and 21%, respectively. The proportion of outpatient visits attributable to host community members in Africa varied significantly from about one percent on average in Djibouti and Rwanda to as high as 30% or greater in Sudan and Uganda. In many settlements in Uganda, the proportion of outpatient visits attributable to host community members was higher than the proportion attributable to refugees. In addition, there is a statistically significant difference in the proportion of new OPD visits attributable to host nationals between Asia and Africa (an average of 18% higher in Africa).

**Table 3:** Mean Proportion of New Outpatient Department Visits per Month by Refugees vs. Host Nationals, 2008-2009

		Refugee		Host National		Ref - Host Difference p Value*
			
Region/Country/Camp		Pct *	95% CI*	Pct *	95% CI*	
**Africa**		**78.9**	**73.7,84.2**	**21.1**	**15.8,26.3**	**< .001**
**Burundi**		**90.8**	**87.4,94.2**	**9.2**	**5.8,12.6**	**< .001**
Bwagiriza		88.9	78.6,99.1	11.1	0.9,21.4	
Gasorwe		91.5	85.2,97.9	8.5	2.1,14.8	
Gihinga		93.6	85.1,102.1	6.4	-2.1,14.9	
Musasa		89.3	83.9,94.6	10.7	5.4,16.1	
**Cameroon**	Langui	**96.7**	**91.1,102.2**	**3.3**	**-2.2,8.9**	**< .001**
**Chad**		**88.1**	**85.9,90.2**	**11.9**	**9.8,14.1**	**< .001**
Amboko		98.9	98.1,99.7	1.1	0.3,1.9	
Amnabak		85.0	81.7,88.4	15.0	11.6,18.3	
Bredjing		95.3	93.5,97.1	4.7	2.9,6.5	
Djabal		94.5	90.8,98.1	5.5	1.9,9.2	
Dosseye		88.3	86.5,90.1	11.7	9.9,13.5	
Farchana		70.2	63.1,77.3	29.8	22.7,36.9	
Gaga		87.1	86.4,87.9	12.9	12.1,13.6	
Gondje		99.1	98.1,100.2	0.9	-0.2,1.9	
Goz Amer		90.3	88.3,92.2	9.7	7.8,11.7	
Kounoungou		84.3	82.5,86.1	15.7	13.9,17.5	
Mile		84.1	81.5,86.7	15.9	13.3,18.5	
Moula		93.3	91.3,95.3	6.7	4.7,8.7	
Oure Cassoni		84.4	81.9,86.9	15.6	13.0,18.1	
Treguine		84.0	80.5,87.5	16.0	12.5,19.5	
Yaroungou		81.6	78.5,84.8	18.4	15.2,21.5	
**Djibouti**	Ali Adde	**98.8**	**98.2,99.3**	**1.2**	**0.7,1.8**	**< .001**
**Ethiopia**		**85.4**	**73.7,97.1**	**14.6**	**2.9,26.3**	**< .001**
Awbarre		98.3	97.6,99.0	1.7	1.0,2.4	
Fugnido		93.8	92.9,94.7	6.2	5.3,7.1	
Kebribeyah		91.4	90.1,92.7	8.6	7.3,9.9	
Sherkole		63.3	52.9,73.7	36.7	26.3,47.1	
Shimelba		72.0	67.8,76.1	28.0	23.9,32.2	
**Guinea**	Kouankan II	**94.8**	**91.2,98.5**	**5.2**	**1.5,8.8**	**< .001**
**Kenya**		**97.2**	**91.9,102.5**	**2.8**	**-2.5,8.1**	**< .001**
Dagahaley		99.7	99.3,100.1	0.3	-0.1,0.7	
Hagadera		99.9	99.9,100.0	0.1	0.0,0.1	
Ifo		99.9	99.9,100.0	0.0	0.0,0.1	
Kakuma		87.2	86.1,88.4	12.8	11.6,13.9	
**Rwanda**		**99.99**	**99.96,100**	**0.01**	**0.0,.03**	**< .001**
Gihembe		99.98	99.9,100	0.0	0.0,0.1	
Kiziba		91.1	88.7,93.6	8.9	6.4,11.3	
Nyabiheke		100		0		
**Sudan**		**64.3**	**53.5,75.0**	**35.7**	**25.0,44.5**	**< .01**
Abuda		56.8	51.8,61.8	43.2	38.2,48.2	
Fau 5		38.4	35.1,41.8	61.6	58.2,64.9	
Girba		57.8	56.1,59.4	42.2	40.6,43.9	
Kilo 26		66.5	62.0,70.9	33.5	29.1,38.0	
Shagarab I II III		94.1	91.5,96.6	5.9	3.3,8.5	
Suki		36.7	35.4,38.0	63.3	62.0,64.6	
Um Gargour		82.9	68.6,97.1	17.1	2.9,31.4	
Wad Sharifey		68.6	65.4,71.8	31.4	28.2,34.6	
**Tanzania**		**93.3**	**91.6,95.0**	**6.7**	**5.0,8.4**	**< .001**
Lugufu		95.0	94.0,96.1	5.0	3.9,6.0	
Lukole		82.9	79.1,86.6	17.1	13.4,20.9	
Mtabila		94.9	94.2, 95.6	5.1	4.4,5.8	
Nduta		95.7	94.1,97.2	4.3	2.8,5.9	
Nyarugusu		92.7	91.7,93.6	7.3	6.4,8.3	
**Uganda**		**44.1**	**33.8,54.4**	**55.9**	**45.6,66.2**	**< .26**
Adjumani		29.8	16.1,43.4	70.2	56.6,83.9	
Ikafe		12.7	-3.8,29.1	87.4	70.9,103.8	
Imvepi		30.7	19.0,42.4	69.3	57.6,81.0	
Kiryandongo		56.9	53.2,60.6	43.1	39.4,46.8	
Kyaka II		63.5	60.0,67.1	36.5	32.9,40.0	
Kyangwali		54.0	48.7,59.3	46.0	40.7,51.3	
Madi Okollo		41.6	2.7,80.4	58.4	19.6,97.3	
Nakivale		89.7	85.8,93.7	10.3	6.3,14.2	
Oruchinga		27.6	19.5,35.7	72.4	64.3,80.5	
Palorinya		33.8	15.3,52.4	66.2	47.6,84.7	
Rhino		20.8	12.1,29.4	79.2	70.6,87.9	
**Yemen**	**Kharaz**	**69.7**	**65.9,73.5**	**30.3**	**26.5,34.1**	**< .001**
**Zambia**		**88.5**	**82.5,94.5**	**11.5**	**5.5,17.5**	**< .001**
Kala		92.0	90.4,93.6	8.0	6.4,9.6	
Maheba		76.1	71.3,80.9	23.9	19.1,28.7	
Mayukwayukwa		85.5	80.5,90.6	14.5	9.4,19.5	
Mwange		98.5	98.0,99.1	1.5	0.9,2.0	
**Asia**		**97.6**	**96.8,98.4**	**2.4**	**1.6,3.2**	**< .001**
**Bangladesh**		**97.4**	**96.0,98.9**	**2.6**	**1.1,4.0**	**< .001**
Kutupalong		98.2	95.3,101.1	1.8	-1.1,4.7	
Nayapara		96.8	96.0,97.6	3.2	2.4,4.0	
**Nepal**		**97.8**	**96.4,99.2**	**2.2**	**0.8,3.6**	**< .001**
Beldangi I		99.4	99.1,99.7	0.6	0.3,0.9	
Beldangi II		99.9	99.95,100	0.0	0.0,0.0	
Beldangi II ext		99.8	99.7,99.9	0.2	0.1,0.3	
Goldhap		97.8	97.5,98.1	2.2	1.9,2.5	
Khudunabari		94.5	93.6,95.3	5.5	4.7,6.4	
Sanishare		99.9	99.8,99.9	0.1	0.1,0.2	
Timai		93.9	93.1,94.7	6.1	5.3,6.9	
**Thailand**		**97.5**	**96.3,98.6**	**2.5**	**1.4,3.7**	**< .001**
Ban Don Yang		96.9	95.6,98.1	3.1	1.9,4.4	
Ban Mae Surin		99.9	99.9,99.9	0.0	0.0,0.1	
Ban Mai Nai Soi		99.9	99.9,100.0	0.0	0.0,0.0	
Mae La		96.4	95.9,97.0	3.6	3.0,4.1	
Mae La Oon		97.3	96.9,97.8	2.7	2.2,3.1	
Mae Ra Ma Luang		98.3	98.0,98.5	1.7	1.5,2.0	
Nu Poh		90.2	89.1,91.3	9.8	8.8,10.9	
Tham Hin		99.9	99.8,99.9	0.1	0.1,0.2	
Umpiem Mai		99.3	99.2,99.5	0.7	0.5,0.8	
**All Regions**		**82.9**	**78.5,87.3**	**17.1**	**12.7,21.5**	**< .001**
**Asia - Africa Differential (p-value)**		**18.6**	**9.2,28.0**			
		**(p < .001)**				

* Values, Confidence Intervals and Significance are based on Generalized Estimating Equations, population-averaged model (Std. Err. adjusted for clustering on Camp)

#### Distribution of gender among refugee populations

Table 4 also shows the proportion of the settlement population that is female (among refugees only). Across all settlements reporting to the UNHCR database, the percent of the refugee population that is female was about the same as the male population; there was no statistically significant difference between the size of the male and female populations in refugee settlements overall. There was some variation, however, within and between regions. Asian settlements, on average, have a slightly higher percentage of males than females, except in Bangladesh. While most of the African settlements had slightly more female refugees than males, Cameroon, Ethiopia, and Kenya have the opposite relationship.

**Table 4:** Percent of New Outpatient Department Visits by Females, Refugee vs Host Country Patients, 2008-2009

		All		Refugee			Host			Pct OPD Female Ref - Host Difference p Value*
			
Region/Country/Camp		Percent OPD Visits Female*	95% CI*	Pct. Refugee Pop. Female *	Pct. OPD Visits Female*	95% CI*	National Pct Pop Female **	Pct. OPD Visits Female*	95% CI*	
**Africa**		**54.4**	**53.9,54.9**	**51.1**	**54.8**	**54.4,55.3**	**50**	**51.7**	**50.5,52.8**	**p < .001**
**Burundi**		**54.2**	**53.0,55.4**	**51.2**	**53.9**	**52.9,55.0**	**51**	**53.0**	**48.9,57.2**	
Bwagiriza		53.1	51.7,54.5	51.1	53.2	51.9,54.5		42.4	26.0,58.9	
Gasorwe		54.9	53.6,56.2	52.2	54.3	53.5,55.1		54.1	46.3,62.0	
Gihinga		56.2	54.8,57.5	50.6	56.0	54.8,57.2		56.2	50.8,61.6	
Musasa		52.2	49.9,54.4	50.5	51.8	50.0,53.6		53.9	49.6,58.3	
**Cameroon**	Langui	**51.6**	**49.6,53.6**	**48.8**	**51.7**	**49.6,53.9**	**50**	**45.5**	**32.9,58.2**	
**Chad**		**53.9**	**53.2,54.6**	**54.9**	**54.4**	**53.7,55.1**	**50**	**48.4**	**46.1,50.7**	**p < .001**
Amboko		54.6	51.4,57.8	53.5	54.9	51.6,58.1		35.5	26.5,44.5	p < .001
Amnabak		55.1	54.0,56.2	61.3	55.1	53.9,56.3		54.8	52.3,57.2	
Bredjing		51.1	48.8,53.5	54.2	52.5	51.4,53.6		34.3	2,8,65.9	
Djabal		52.6	51.5,53.7	54.4	53.0	51.7,54.2		45.7	42.0,49.4	p < .001
Dosseye		57.7	56.8,58.6	54.8	59.2	58.6,59.7		46.3	42.1,50.6	p < .001
Farchana		48.0	45.1,50.8	55.3	49.2	45.9,52.4		45.8	43.7,47.9	p < .05
Gaga		52.7	51.6,53.9	54.4	53.0	51.9,54.1		51.3	49.3,53.4	
Gondje		53.2	51.0,55.3	51.6	53.3	51.2,55.4		39.5	26.7,52.3	p < .05
Goz Amer		52.7	51.3,54.1	53.3	53.0	51.6,54.3		49.3	47.8,50.7	p < .001
Kounoungou		55.4	54.4,56.3	56.8	55.1	54.3,55.9		56.6	54.4,58.8	
Mile		56.7	55.3,58.2	56.2	57.4	56.1,58.7		52.8	50.7,54.9	p < .001
Moula		53.0	51.3,54.7	49.5	53.4	51.3,55.5		44.8	37.3,52.3	
Oure Cassoni		57.7	55.3,60.1	60.2	58.4	55.1,61.6		54.3	51.9,56.7	
Treguine		49.3	48.4,50.1	51.3	49.5	48.4,50.5		48.4	46.6,50.2	
Yaroungou		54.7	52.6,56.9	53.2	56.0	52.3,59.7		48.1	41.2,55.0	
**Djibouti**	Ali Adde	**56.2**	**55.2,57.2**	**50.8**	**56.3**	**55.2,57.4**	**50**	**46.4**	**39.6,53.2**	**p < .01**
**Ethiopia**		**52.6**	**49.5,55.7**	**46.2**	**52.3**	**48.7,56.0**	**50**	**50.3**	**48.6,51.9**	
Awbarre		57.7	56.0,59.4	50.9	57.7	56.1,59.4		52.9	47.8,58.0	
Fugnido		56.3	55.0,57.7	54.9	56.8	55.4,58.3		49.3	46.4,52.2	p < .001
Kebribeyah		54.8	53.8,55.8	50.4	55.0	54.0,55.9		53.0	50.2,55.8	
Sherkole		49.2	45.1,53.2	45.2	48.4	42.9,54.0		49.3	47.8,50.9	
Shimelba		42.4	41.7,43.1	28.3	40.8	40.2,41.4		46.9	43.9,50.0	p < .001
**Guinea**	Kouankan II	**56.5**	**54.2,58.7**	**53.2**	**56.7**	**54.2,59.1**	**50**	**54.9**	**51.6,58.2**	
**Kenya**		**50.3**	**49.3,51.2**	**47.8**	**50.3**	**49.3,51.3**	**50**	**49.2**	**40.5,58.0**	
Dagahaley		51.3	50.5,52.2	49.4	51.3	50.5,52.2		63.3	33.7,93.0	
Hagadera		51.7	50.6,52.7	48.7	51.7	50.7,52.7		47.5	32.9,62.1	
Ifo		50.9	48.6,53.2	48.9	50.9	48.6,53.2		42.7	18.2,67.2	
Kakuma		47.1	46.6,47.7	44.1	47.3	46.5,48.1		46.6	44.7,48.5	
**Rwanda**		**56.4**	**55.0,57.9**	**55.2**	**56.4**	**55.0,57.9**	**52**	**--**	**--**	
Gihembe		56.2	54.8,57.6	54.9	56.2	54.8,57.6		--	--	
Kiziba		58.3	56.3,60.3	55.0	58.3	56.4,60.2		57.9	54.2,61.6	
Nyabiheke		54.3	51.9,56.8	55.9	54.3	51.9,56.8		--	--	
**Sudan**		**55.6**	**54.0,57.3**	**50.0**	**57.3**	**56.6,58.0**	**50**	**52.4**	**46.7,58.0**	
Abuda		58.3	55.1,61.5	48.7	60.5	59.0,62.0		55.7	50.6,60.7	p < .05
Fau 5		52.8	51.3,54.3	54.6	56.5	54.9,58.0		50.8	48.7,52.9	p < .001
Girba		54.6	46.3,62.9	50.2	58.0	57.2,58.8		49.5	29.6,69.4	
Kilo 26		53.7	47.4,59.9	45.2	55.2	54.6,55.8		49.5	30.5,68.5	
Shagarab I II III		57.6	53.1,62.2	49.5	58.2	54.8,61.6		56.0	30.6,81.5	
Suki		56.3	54.2,58.4	48.5	55.7	53.8,57.7		56.6	53.2,60.1	
Um Gargour		54.8	51.6,58.0	47.7	56.6	55.7,57.5		46.8	28.9,64.8	
Wad Sharifey		56.8	56.0,57.5	55.9	57.6	57.0,58.2		55.2	53.0,57.4	p < .05
**Tanzania**		**52.8**	**51.7,53.9**	**50.7**	**52.9**	**51.8,54.0**	**50**	**51.2**	**49.0,53.3**	**p < .05**
Lugufu		49.4	47.5,51.2	51.0	49.3	47.4,51.2		48.9	43.5,54.3	
Lukole		55.0	54.2,55.7	49.4	55.8	54.9,56.7		51.0	50.9,51.1	p < .001
Mtabila		55.3	54.8,55.8	50.5	55.3	54.8,55.8		55.5	53.0,58.0	
Nduta		54.8	53.6,56.0	50.7	54.8	53.5,56.1		53.4	50.3,56.5	
Nyarugusu		51.5	50.5,52.5	51.1	51.8	50.8,52.8		47.2	44.9,49.5	p < .001
**Uganda**		**57.1**	**56.2,58.0**	**50.2**	**57.5**	**56.5,58.5**	**50**	**56.6**	**55.4,57.7**	
Adjumani		57.0	55.3,58.7	51.3	58.8	57.2,60.6		55.8	53.3,58.3	
Ikafe		55.3	53.2,57.4	46.0	58.3	53.7,62.9		53.6	49.8,57.3	
Imvepi		54.4	50.1,58.7	51.2	55.7	48.7,62.6		55.5	52.0,59.1	
Kiryandongo		56.7	54.8,58.7	49.8	57.6	55.6,59.6		56.1	53.3,59.0	
Kyaka II		56.2	54.1,58.2	50.5	54.5	53.2,55.9		58.0	54.3,61.7	
Kyangwali		56.6	54.9,58.3	50.3	58.0	56.8,59.3		55.1	52.6,57.7	p < .001
Madi Okollo		60.9	56.3,65.6	49.6	55.1	50.8,59.4		59.6	52.6,66.7	
Nakivale		56.4	54.2,58.7	51.1	56.2	53.8,58.7		56.5	53.8,59.3	
Oruchinga		57.7	54.5,60.9	49.7	57.7	56.7,58.7		57.0	52.5,61.6	
Palorinya		59.8	56.7,62.9	51.8	61.9	59.6,64.2		58.2	54.2,62.1	p < .01
Rhino		57.1	52.7,61.5	48.0	57.4	54.1,60.8		56.3	50.4,62.1	
**Yemen**	Kharaz	**53.6**	**51.5,55.7**	**50.9**	**53.3**	**51.2,55.4**	**49**	**53.8**	**52.0,55.7**	
**Zambia**		**53.9**	**52.5,55.3**	**49.9**	**54.3**	**52.9,55.7**	**50**	**51.4**	**49.2,53.6**	p < .01
Kala		50.9	49.6,52.2	50.6	51.4	50.1,52.7		47.6	43.4,51.7	
Maheba		52.6	50.4,54.8	48.8	53.1	50.7,55.6		49.2	45.5,52.9	p < .05
Mayukwayukwa		57.6	55.5,59.7	49.7	58.2	55.8,60.5		55.1	53.8,56.5	p < .01
Mwange		54.4	53.4,55.4	50.6	54.4	53.3,55.4		53.5	48.3,58.6	
**Asia**		**53.3**	**52.9,53.8**	**49.4**	**53.4**	**52.9,53.9**	**50**	**48.5**	**46.4,50.5**	**p < .001**
**Bangladesh**		**53.3**	**51.9,54.6**	**51.5**	**53.7**	**52.2,55.1**	**49**	**37.4**	**32.5,42.3**	**p < .001**
Kutupalong		52.8	51.0,54.7	51.2	53.2	51.1,55.2		37.1	28.5,45.7	p < .001
Nayapara		53.7	51.8,55.6	51.9	54.2	52.4,56.1		37.6	32.9,42.4	p < .001
**Nepal**		**54.1**	**53.7,54.5**	**49.2**	**54.2**	**53.8,54.5**	**50**	**50.2**	**47.0,53.4**	**p < .05**
Beldangi I		54.0	53.2,54.8	49.2	54.0	53.2,54.8		51.8	44.4,59.2	
Beldangi II		54.4	52.9,56.0	49.2	54.4	52.9,56.0		59.1	47.3,70.8	
Beldangi II ext		54.4	53.5,55.4	49.0	54.4	53.5,55.4		51.3	39.9,62.7	
Goldhap		53.9	53.0,54.8	48.8	53.9	53.0,54.9		51.8	49.9,53.8	
Khudunabari		54.5	53.5,55.5	49.8	54.6	53.6,55.7		52.2	51.0,53.4	p < .01
Sanishare		54.2	53.6,54.9	49.3	54.2	53.6,54.9		39.6	27.9,51.3	p < .05
Timai		53.2	52.3,54.1	49.0	53.3	52.3,54.2		51.9	50.8,52.9	p < .05
**Thailand**		**52.7**	**51.9,53.5**	**49.1**	**52.7**	**52.0,53.5**	**51**	**50.1**	**47.5,52.7**	**p < .05**
Ban Don Yang		54.3	53.3,55.2	51.0	54.3	53.3,55.3		55.0	50.2,59.7	
Ban Mae Surin		53.3	52.2,54.4	48.4	53.3	52.3,54.4		52.1	12.3,91.8	
Ban Mai Nai Soi		49.5	48.7,50.3	48.2	49.5	48.7,50.3		--	--	
Mae La		50.5	49.1,51.9	49.3	50.4	49.1,51.8		50.8	48.4,53.2	
Mae La Oon		49.6	45.5,53.8	49.1	49.7	45.5,53.9		45.5	41.8,49.3	p < .01
Mae Ra Ma Luang		54.4	53.2,55.5	49.9	54.5	53.3,55.6		49.2	47.1,51.3	p < .001
Nu Poh		53.7	52.7,54.7	48.2	53.8	52.8,54.8		52.8	51.7,53.9	
Tham Hin		53.9	52.9,54.9	48.9	53.9	52.9,54.9		46.7	33.7,59.7	
Umpiem Mai		55.3	54.4,56.2	48.5	55.3	54.5,56.2		49.9	43.3,56.3	
**All Regions**		**54.1**	**53.8,54.5**	**50.7**	**54.5**	**54.1,54.9**	**50**	**50.9**	**49.9,51.9**	**p < .001**
**Asia - Africa Differential (p-value)**		**-1.1** **(p < .05)**	**-2.0,-0.2**	**-1.7 (p < .10)**	**-1.4**	**-2.3,-0.6 (p < .01)**	**0**	**-3.2**	**-5.5,-0.9** **(p < .01)**	

* Values, Confidence Intervals and Significance are based on Generalized Estimating Equations, population-averaged model (Std. Err. adjusted for clustering on Camp); only p-values significant to the .05 level or less are provided.

** Source: World Bank, Health, Nutrition and Population database estimates for 2008 http://databank.worldbank.org.

Note that the UNHCR database does not include information on the size and distribution of the host populations living near the refugee settlements reporting to the database. For this reason, we included national estimates of the size of the female population for host countries. Asian and African countries included in the database, on average, have about the same number of males and females. There are no striking differences between the percent of refugee settlement populations that are female, and the national estimates of the percent of host country populations that are female.

#### New OPD Visits per Month by Gender

Table 4 shows mean proportion of new visits in a month attributable to females. In all but one country (Chad), the proportion of new OPD visits per month attributable to female refugees was higher than the female proportion of the refugee population.

In a majority of African countries, the proportion of new OPD visits per month attributable to host national females was higher than national estimates of the female population in the host country. In Asia, this happened only in Bangladesh; in the other two Asian countries, the proportion of new OPD visits per month attributable to host national females was lower than national estimates of the female population in the host country.

The proportion of new OPD visits per month attributable to female refugees was also higher than the proportion of new OPD visits attributable to females among host nationals, with the exception of Yemen and Thailand.

The proportion of new OPD visits per month attributable to women (among both refugee and host nationals) was higher in African settlements than in Asian settlements. This regional difference was greater among host nationals than among refugees.

### Proportion of New Outpatient Diagnoses per Month

#### Proportion of new outpatient diagnoses by age

Table 5 depicts the mean proportion of new outpatient diagnosis each month attributable to children under five years of age. Table 5 also compares this same proportion between refugees and host nationals utilizing settlement outpatient services. Because the UNHCR's Health Information System database does not document new visits by age group, we have included analysis of new outpatient diagnoses to allow us to look at age patterns in use of services. By looking at diagnoses, we understand that one person may have multiple diagnoses on a single visit; there is not a one to one ratio between visits and diagnoses. The database available only allows for age-specific analysis for two groups: (1) under five years; or, (2) five years of age or higher.

**Table 5:** Percent of Outpatient Department Diagnoses by Children Less than Five Years of Age (U5), Refugee vs Host Country Patients, 2008-2009

		All		Refugee			Host			Pct OPD U5 Ref - Host Difference p Value*
			
Region/Country/Camp		Percent OPD Diagnoses U5*	95% CI*	Pct. Refugee Pop. U5 *	Pct. OPD Diagnoses U5*	95% CI*	National Pct. Pop. U5 **	Pct. OPD Diagnoses U5*	95% CI*	
**Africa**		**38.6**	**37.6,39.5**	**16.9**	**37.4**	**36.3,38.5**	**16.2**	**39.4**	**38.2,40.6**	**p < .001**
**Burundi**		**39.8**	**37.3,42.4**	**19.4**	**40.7**	**38.0,43.4**	**14.3**	**28.2**	**24.3,32.2**	**p < .001**
Bwagiriza		38.3	29.5,47.0	23.4	38.8	30.3,47.2		23.4	7.6,39.3	p < .01
Gasorwe		40.3	39.1,41.6	22.8	41.9	40.5,43.2		23.5	17.7,29.3	p < .001
Gihinga		35.8	33.9,37.7	14.9	36.5	36.5,38.5		27.5	22.9,32.1	p < .01
Musasa		41.9	35.0,48.7	18.6	42.3	35.2,49.5		37.5	32.2,42.7	p < .001
**Cameroon**	Langui	**26.6**	**21.1,32.0**	**18.5**	**26.5**	**21.1,31.9**	**15.8**	**29.6**	**20.2,38.9**	
**Chad**		**41.7**	**40.2,43.3**	**18.4**	**41.9**	**40.3,43.6**	**18.2**	**39.4**	**37.1,41.8**	
Amboko		41.6	31.2,51.9	12.2	41.6	31.2,52.1		28.5	16.7,40.3	
Amnabak		36.3	34.1,38.5	23.3	36.0	33.4,38.6		40.6	37.2,44.0	
Bredjing		41.4	37.9,44.9	19.0	40.2	37.8,42.7		53.5	37.3,69.7	
Djabal		39.6	36.7,42.5	21.2	39.7	36.9,42.4		37.3	29.4,45.3	
Dosseye		40.8	37.5,44.1	19.7	38.4	34.8,42.1		53.5	45.1,61.9	p < .01
Farchana		44.1	39.8,48.3	17.2	45.2	41.0,49.5		40.1	36.8,43.4	p < .001
Gaga		44.7	41.3,48.2	20.9	45.8	42.0,49.6		37.7	30.7,44.8	p < .05
Gondje		43.2	30.9,55.4	11.3	43.3	31.1,55.5		26.5	12.2,40.7	
Goz Amer		43.7	40.1,47.3	22.2	44.2	39.8,48.5		41.2	37.9,44.6	
Kounoungou		40.2	38.7,41.8	17.4	41.2	39.6,42.8		35.8	30.6,41.0	
Mile		41.7	38.6,44.8	17.4	43.1	39.0,47.2		35.0	30.2,39.8	
Moula		37.5	23.7,51.2	25.0	38.7	24.0,53.4		26.6	22.8,30.3	p < .01
Oure Cassoni		41.7	39.5,43.9	15.6	42.0	39.4,44.5		41.4	35.7,47.1	
Treguine		43.2	39.6,46.9	19.1	44.3	40.1,48.6		38.7	33.3,44.1	
Yaroungou		43.9	39.6,48.2	18.0	42.2	36.4,48.1		49.6	44.4,54.9	p < .05
**Djibouti**	Ali Adde	**34.5**	**31.0,38.1**	**16.1**	**34.5**	**31.0,38.1**	**13.5**	**34.0**	**16.3,51.7**	
**Ethiopia**		**41.8**	**39.9,43.7**	**17.7**	**41.4**	**39.3,43.4**	**16.5**	**40.6**	**35.1,46.0**	
Awbarre		47.7	44.7,50.7	19.7	48.3	45.2,51.5		41.0	27.4,54.6	
Fugnido		40.9	37.7,44.1	23.8	42.1	38.6,45.6		27.2	16.4,38.0	p < .05
Kebribeyah		38.3	36.3,40.3	20.6	37.8	35.7,39.8		43.1	38.1,48.1	p < .05
Sherkole		39.5	36.5,42.6	18.1	38.1	35.5,40.7		42.2	36.2,48.7	
Shimelba		43.2	40.0,46.3	9.0	40.7	37.0,44.4		48.9	45.1,52.8	p < .01
**Guinea**	Kouankan II	**28.5**	**26.5,30.6**	**14.2**	**27.8**	**26.1,29.6**	**16.7**	**35.7**	**21.4,49.9**	
**Kenya**		**39.5**	**37.8,41.2**	**15.4**	**39.3**	**37.4,41.1**	**16.9**	**39.7**	**33.5,46.0**	
Dagahaley		40.6	35.4,45.7	17.2	40.6	35.4,45.7		28.1	15.2,40.9	p < .001
Hagadera		39.0	37.4,40.6	14.9	39.0	37.4,40.5		40.5	19.7,61.3	
Ifo		41.7	39.7,43.8	15.4	41.7	39.7,43.8		33.9	16.7,51.1	
Kakuma		36.5	33.9,39.1	14.3	35.5	32.6,38.3		43.6	38.8,48.4	p < .01
**Rwanda**		**37.4**	**34.4,40.5**	**20.1**	**37.4**	**34.4,40.5**	**17.0**	**--**		
Gihembe		32.8	30.7,34.9	17.3	32.8	30.7,34.9		--		
Kiziba		38.6	34.2,43.0	21.7	38.6	34.2,43.1		37.8	31.9,43.7	
Nyabiheke		41.0	35.2,46.7	21.4	41.0	35.2,46.7		--		
**Sudan**		**30.2**	**27.1,33.3**	**9.2**	**27.1**	**25.2,29.0**	**14.1**	**34.9**	**30.7,39.1**	**p < .001**
Abuda		27.6	22.9,32.3	9.2	25.1	23.0,27.1		33.9	22.9,44.9	p < .05
Fau 5		42.8	40.1,45.4	9.5	34.4	31.3,37.5		47.8	44.0,51.6	p < .001
Girba		29.6	27.3,31.9	7.9	27.9	26.0,29.9		33.8	25.7,41.8	
Kilo 26		18.9	16.5,21.3	11.1	16.9	14.0,19.8		25.7	15.2,36.1	
Shagarab I II III		27.6	25.6,29.7	14.8	27.3	25.4,29.1		29.5	20.7,38.2	
Suki		36.8	35.4,38.1	4.5	31.9	28.3,35.4		39.6	35.6,43.5	
Um Gargour		29.7	27.1,32.3	11.2	28.1	26.0,30.4		37.6	34.0,41.1	p < .001
Wad Sharifey		30.0	25.3,34.7	5.1	27.7	26.1,29.1		34.1	23.3,44.9	
**Tanzania**		**41.8**	**38.1,45.4**	**20.3**	**41.5**	**37.8,45.2**	**17.8**	**44.2**	**42.1,46.2**	**p < .05**
Lugufu		50.0	47.7,52.2	20.0	50.2	48.0,52.4		46.6	43.2,50.0	p < .05
Lukole		43.8	39.3,48.2	24.9	42.6	37.2,48.0		48.4	47.8,49.0	p < .05
Mtabila		41.5	39.8,43.2	20.0	41.3	39.5,43.0		44.0	42.9,46.9	p < .01
Nduta		32.2	27.0,37.3	20.0	31.8	27.1,36.5		39.7	26.3,53.1	
Nyarugusu		39.6	38.3,40.9	19.8	39.3	37.8,40.7		43.4	41.6,45.3	p < .01
**Uganda**		**37.8**	**36.4,39.2**	**17.1**	**33.6**	**31.9,35.3**	**19.5**	**40.8**	**38.9,42.8**	**p < .001**
Adjumani		41.8	39.9,43.8	14.3	35.2	34.0,36.4		44.7	42.3,47.2	p < .001
Ikafe		45.8	42.8,48.7	13.4	40.3	35.0,45.5		46.6	43.2,49.9	p < .001
Imvepi		34.6	32.2,37.0	10.9	24.1	21.6,26.7		41.0	35.6,46.4	p < .001
Kiryandongo		36.3	34.4,38.3	19.0	34.3	31.2,37.5		38.5	32.7,44.4	
Kyaka II		42.6	38.9,46.3	24.8	41.3	37.4,45.3		45.3	39.7,50.8	
Kyangwali		38.9	36.2,41.5	19.9	35.6	33.9,37.3		42.8	38.3,47.3	p < .001
Madi Okollo		34.5	31.0,38.0	15.7	30.0	26.5,33.4		37.8	30.7,44.9	p < .001
Nakivale		31.6	27.7,35.6	19.2	31.6	27.4,35.7		35.9	29.0,42.8	
Oruchinga		35.1	30.9,39.3	21.5	30.9	26.1,35.7		36.7	30.3,43.2	
Palorinya		38.1	34.3,42.0	15.1	38.3	35.2,41.5		38.5	33.0,44.0	
Rhino		38.7	32.2,45.2	12.4	27.5	23.8,31.1		42.0	33.1,50.8	p < .001
**Yemen**	**Kharaz**	**40.7**	**38.1,43.4**	**19.7**	**40.4**	**36.7,44.2**	**16.3**	**41.2**	**39.3,43.2**	
**Zambia**		**40.6**	**38.0,43.2**	**19.7**	**40.8**	**38.3,43.3**	**18.1**	**38.8**	**35.0,42.5**	
Kala		40.9	38.5,43.4	20.0	40.6	38.2,43.0		44.8	41.0,48.5	p < .05
Maheba		39.4	35.6,43.2	19.2	39.7	35.7,43.7		39.4	35.8,43.1	
Mayukwayukwa		36.1	34.2,38.0	21.4	36.8	34.4,39.2		30.7	27.6,33.8	p < .01
Mwange		46.9	41.5,52.4	18.0	47.0	41.5,52.5		40.9	31.5,50.3	
**Asia**		**30.0**	**28.9,31.1**	**12.1**	**30.1**	**29.0,31.1**	**9.8**	**24.4**	**21.5,27.2**	**p < .01**
**Bangladesh**		**34.8**	**32.9,36.7**	**18.5**	**35.0**	**33.2,36.9**	**10.4**	**22.8**	**15.1,30.5**	**p < .01**
Kutupalong		35.4	32.0,38.8	19.0	35.5	32.2,38.9		23.7	15.0,32.4	p < .01
Nayapara		34.1	32.3,35.9	18.2	34.5	32.9,36.2		22.3	8.8,35.7	
**Nepal**		**30.9**	**29.6,32.2**	**8.0**	**30.8**	**29.4,32.1**	**12.3**	**27.5**	**23.5,31.4**	
Beldangi I		32.3	30.5,34.1	8.6	32.3	30.5,34.1		30.8	23.6,38.0	
Beldangi II		28.6	26.3,30.9	7.2	28.6	26.3,30.9		10.0	-3.3,23.4	p < .01
Beldangi II ext		30.1	28.4,31.8	8.1	30.1	28.4,31.8		18.2	8.7,27.7	p < .01
Goldhap		32.6	29.7,35.4	8.1	32.5	29.6,35.4		34.7	29.6,39.8	
Khudunabari		26.5	25.5,27.4	6.8	25.7	24.7,26.8		38.7	33.5,44.0	p < .001
Sanishare		36.3	34.7,38.0	8.0	36.4	34.8,38.0		14.3	4.5,24.2	p < .001
Timai		29.3	28.1,30.4	8.7	29.1	27.9,30.3		32.4	26.9,37.8	
**Thailand**		**28.2**	**26.6,29.6**	**13.5**	**28.3**	**26.9,29.8**	**7.2**	**18.4**	**15.4,21.5**	**p < .001**
Ban Don Yang		24.7	23.8,25.7	14.9	25.0	24.0,26.0		18.0	12.3,23.7	p < .01
Ban Mae Surin		26.5	25.5,27.5	13.8	26.5	25.5,27.5		8.3	-6.3,23.0	p < .001
Ban Mai Nai Soi		39.2	35.9,42.6	12.1	39.2	35.9,42.6		--		
Mae La		24.6	23.4,25.8	11.1	24.8	23.6,26.0		16.1	9.0,23.2	p < .001
Mae La Oon		28.7	26.9,30.4	13.2	28.9	27.2,30.7		19.8	10.9,28.7	p < .05
Mae Ra Ma Luang		26.4	25.3,27.5	15.1	26.6	25.4,27.7		16.2	7.1,25.4	p < .05
Nu Poh		26.0	25.1,26.9	12.0	26.8	25.8,27.8		17.1	10.2,24.1	p < .001
Tham Hin		30.5	28.7,32.4	17.1	30.5	28.7,32.4		16.4	3.6,29.2	p < .05
Umpiem Mai		25.9	24.8,27.0	11.3	25.9	24.8,27.0		19.4	5.3,33.6	
**All Regions**		**36.5**	**35.0,37.9**	**15.7**	**35.6**	**34.7,36.6**	**13.9**	**36.2**	**34.8,37.6**	
**Asia - Africa Differential**		**-8.6**	**-11.5,-5.7**	**-5.0**	**-7.3**	**-9.3,-5.4**	**-6.4**	**-15.0**	**-17.8,-12.3**	
			**p < .001**	**p < .001**		**p < .001**			**p < .001**	

* Values, Confidence Intervals and Significance are based on Generalized Estimating Equations, population-averaged model (Std. Err. adjusted for clustering on Camp); only p-values significant to the .05 level or less are provided.

** Source: World Bank, Health, Nutrition and Population database estimates for 2008 http://databank.worldbank.org.

Across all settlements reporting to the UNHCR database, the percent of the refugee population that was less than five years of age is 16% on average (Table 5). The average under-five year population for Asia was significantly lower than the overall average at 12%. In general, the Asian population living in refugee settlements was older than the population living African settlements. However, there was considerable variation among countries. For example, Bangladesh, Tanzania, Rwanda, Yemen and Zambia had an average under-five refugee population greater than 19%, while Nepal and Sudan had rates as low as 8-9%. National estimates of the size of the under-five population in host countries are also provided in Table 5 for comparison (this information is not available at the local level for host populations using refugee settlement health services). Across all countries contributing to the database, the estimated under-five population is an average of 14% (weighted for population size of included countries). For African countries, the average is 16%; it is 10% for Asian countries. There is substantial variation between countries in the estimated proportion less than five years of age: from 7% in Thailand to over 19% in Uganda.

#### Proportion of new outpatient diagnoses attributable to children less than five years of age by status (refugee vs. host national)

Although under-fives make up 16% of refugee settlement populations on average, they represent 36% of all outpatient diagnoses among refugees. Very similar, although the national estimates of the size of the under-five population among host countries averages at 14%, under-fives represent 36% of outpatient diagnoses among host nationals.

The proportion of outpatient diagnoses attributable to under-fives among host nationals was slightly higher (39%), on average, than the proportion of outpatient diagnoses attributable to under-fives among refugees (37%). This pattern was consistent across most African countries except for Burundi. In Asia, in constrast, the proportion of outpatient diagnoses attributable to under-fives among host nationals was much lower (24%) than the proportion of outpatient diagnoses attributable to under-fives among refugees (30%). Overall, the proportion of all new outpatient diagnoses attributable to under-fives was lower in Asia (30%) as compared to Africa (39%).

## Discussion

Several studies have compared use of reproductive health and HIV health services by refugees versus host communities. However, there is limited information in the literature about general patterns of use of refugee health facilities by refugees and members of host communities. The availability of a database, that combines reports from the majority of refugee settlements supported by UNHCR and partners, provides a unique opportunity to explore how services differ between gender and age groups, and between refugees and host nationals who utilize the health services of the settlements. The structure of the database also allows us to look at overall patterns and to compare and contrast these patterns between and within regions and countries.

### Utilization rates

Utilization rates among refugees vary between regions. In Africa, the average utilization rate is 1.8. However, in Asia, it is 3.5. Both rates are within the range of 1-4 visits per person per year recommended by SPHERE for the emergency phase. The data in this analysis come from refugee settlements in the post-emergency phase, and therefore the SPHERE standard for emergencies may not be applicable, or may need to vary by region or context. The current SPHERE standard for emergencies of 1-4 visits per person per year appears to be relevant for Asia in the post-emergency phase, but not for Africa. In Africa, a post-emergency standard of 1-2 visits per person per year should be considered.

A few settlements had significant over-utilization rates (> 4 visits per person per year). One question is whether this increased utilization was due to a specific public health problem during the 2008-2009, or if it is due to specific cultural factors or health-seeking behaviors in certain populations. In contrast, some settlements had lower than expected utilization rates. This may suggest inadequate access to settlement health facilities, low quality of settlement health services, and/or the availability of competing health services of higher quality. It may also reflect acute events that restrict refugee access to health services in camps for limited periods. For example, insecurity (e.g. militia attacks in Chad) or natural disasters (e.g. local flooding in Kenya) or a mix may be explanations.

Analysis of gender differences in utilization rates reveals that female refugees utilize outpatient services at a higher rate (visits per person per year) than male refugees. This pattern of higher service utilization among female refugees is consistent across regions and countries. One possible explanation is that women use outpatient services for their own routine care, additional reproductive health needs, and are more likely than men to accompany children who need services [10].

### Distribution of Outpatient Service Users

Overall, the number of refugees using settlement outpatient services is higher than the number of host nationals using the same services. This pattern is expected due to the remote/closed nature of refugee settlements in many countries. This means that---although in principle services are free of charge and accessible to nationals---host populations often prefer to visit host government sites closer by. UNHCR often also invests in local health services in refugee hosting areas (e.g., referral hospitals) which could help promote local access to them instead of services inside settlements. Other possible determinants of health service utilization are the direct and indirect costs of using the service and perceived quality of care [11]. However, the latter determinants are context specific and thus difficult to generalize for all refugee settlement situations.

In Uganda generally, and in some settlements in Sudan, however, the opposite trend is observed. In these special cases, host community members account for more visits to refugee settlement outpatient services than refugees. This may reflect the attention to integrated services for refugees and host nationals in Uganda, especially among settlements near the Sudanese border, that appears in the literature [4,12-14]. In Uganda, for example, refugee settlements are no longer refugee camps. Refugees were integrated into existing villages and health services, some of which already existed and others which were newly created and are available to all. The Ugandan Ministry of Health is now a direct implementing partner of UNHCR in some refugee settlements, and UNHCR entirely handed back services to local districts. No refugee-specific services exist anymore in these places, and therefore it is expected that refugee and host access will be more equitable.

In eastern Sudan, a number of refugee camps are located in remote areas more than 15 km from the nearest national health facility. Therefore, host populations living near to refugee camps prefer to seek care in the refugee health facilities, as they are much closer by walking distance (only 2 - 6 km). Even in areas where national health facilities are available, refugee health facilities are often the preferred choice for host communities as there is a perception that national health services cannot meet the needs of host communities due to inadequate staffing and lack of basic medical supplies. In addition, high prescription and referral costs in national services often act as barriers to access to government services; whilst in comparison these tend to be more heavily subsidized within refugee camps.

The proportion of new OPD visits per month attributable to female refugees was higher than the female proportion of the refugee population (in all but one settlement). Similarly, in most African countries, the proportion of new OPD visits attributable to host national females was higher than national estimates of the proportion of females living in the host country. In Asia, in contrast, this happened only in Bangladesh. In Nepal and Thailand, females use refugee-settlement health services less than would be expected given their relative size of the population.

### Distribution of Diagnoses in Outpatient Services

The proportion of outpatient diagnoses attributable to refugee children less than five years of age accounts for over one third (36%) of all refugee outpatient diagnoses, despite the fact that the under five population makes up only 16% of the overall refugee population in this study. Very similar, although the national estimates of the size of the under-five population among host countries average at 14%, under-fives also represent 36% of outpatient diagnoses among host nationals.

It is generally assumed that under-fives make up about 20% of the population in most emergency settings. In these protracted, post-emergency settings, however, it appears that the under-five population size approximates that of the host countries. For example, in Africa, under-fives represented 16-17% of both the refugee population and the national-level estimate for the host country. In Asia, under-fives represented 12% of the refugee population, and 10% of the national estimate of the host country population. This is probably one explanation for why the proportion of all new outpatient diagnoses attributable to under-fives was lower in Asia (30%) as compared to Africa (39%).

The possible influences on the increased utilization among under-fives proportionate to population size are multi-factorial, such as the following: a child's nutritional status; the mother's knowledge and practice of how to prevent and appropriately manage childhood illness; the social and care environment of the household; and, increased susceptibility to infectious disease along with poor access to adequate water supply, sanitation, and immunizations. These are all potential factors leading to a larger number of diagnoses among these children compared to persons aged five years and above [15].

### Limitations

Because we have no data about the size and distribution of the host populations that are using refugee settlement health facilities, we cannot assess the rate at which this population uses these settlement services. We are limited to observing the following among members of the host communities: (1) the percent of all visits made to the outpatient departments of refugee settlement facilities that are made by members of the host national community; (2) the proportion of these new outpatient visits by host nationals that are made by females vs. males; and (3) the proportion of new outpatient diagnoses by host nationals attributable to under-fives vs. those five years of age and older. The UNCHCR database disaggregates use of health services by only two age groups (under fives and five years and above). This limits how much we can identify differences in utilization by age. There may be variations between settlements in how utilization numbers and population numbers are collected and reported to UNHCR, making it difficult to ensure the validity of comparisons between settlements and countries. Finally, interpretation of the differences in specific settlements, countries and regions is somewhat limited by lack of contextual information in the database to explain these differences.

## Conclusions

The availability of a centralized database of health information across UNHCR-supported refugee settlements is a rich resource that is only recently being utilized for across-settlement analyses. Several conclusions can be made from this initial analysis. As seen in Uganda, when refugee health services are integrated into existing host government services, refugees and locals clearly share these services more. This is good for equity but more work needs to be done to examine how quality of services change during and following integration.

The SPHERE standard for emergencies of 1-4 visits per person per year appears to be relevant for Asia in the post-emergency phase, but not for Africa. In Africa, a post-emergency standard of 1-2 visits per person per year should be considered, where investigation is indicated if the rate in particular settlement is above or below that standard. Why some settlements in the database had utilization rates higher or lower than the expected should be explored.

Although it is often assumed that the size of the female population in refugee settlements is higher than males, we found no statistically significant difference between the size of the male and female populations in refugee settlements overall. With a few exceptions, African settlements tended to have more females, whereas Asian settlements tended to have more males. The data do support the idea, however, that females utilize health services more than males and more than their representative size of the population.

Another assumption---that the under-fives make up 20% of the settlement population during the emergency phase---does not appear to hold for the post-emergency phase. Under-fives made up 17% of the refugee population in Africa, 12% of the population in Asian settlements, and 16% overall. Across both regions, under-fives use health services at a higher proportion than their numbers would suggest (37% of OPD visits vs. representing 16% of the population).

## Competing interests

All authors have received salary support from the UN High Commissioner for Refugees. This salary support has covered implementation of the Health Information System described in this paper and/or for writing this manuscript.

## Authors' contributions

All authors have read and approved the final version of the manuscript. WW wrote key sections of the Methods, Results, Discussions and Conclusions. He also designed and carried out exploratory and statistical analysis. AV wrote key sections of the Background and Discussion and edited the manuscript. HT and SM compiled the data for analysis, helped write the Background, and edited the manuscript. CH and PS edited the manuscript and provided key input into the analysis approach and conclusions.
